# New Approach to Dental Morphometric Research Based on 3D Imaging Techniques

**DOI:** 10.3390/jimaging7090184

**Published:** 2021-09-12

**Authors:** Armen V. Gaboutchian, Vladimir A. Knyaz, Dmitry V. Korost

**Affiliations:** 1 Institute of Medicine, Peoples’ Friendship University (RUDN), 117198 Moscow, Russia; 2 Phystech School of Applied Mathematics and Informatics, Moscow Institute of Physics and Technology (MIPT), 141701 Dolgoprudny, Russia; knyaz@gosniias.ru; 3 State Research Institute of Aviation Systems (GosNIIAS), 125319 Moscow, Russia; 4 Faculty of Geology, Moscow State University, 119991 Moscow, Russia; dkorost@mail.ru

**Keywords:** dental morphology, automated digital odontometry (ADO), morphometrics, micro computed tomography (*μ*CT ), odontometry, imaging techniques, image processing, surface analysis, 3D reconstructions, internal dental morphology

## Abstract

Recent progress in imaging and image processing techniques has provided for improvements in odontological research in a variety of aspects. Thus, the presented method has been developed precisely in order to assess metrically 3D reconstructions of teeth. Rapidly and accurately obtained data of a wide range and appropriate density are sufficient enough for morphometric studies rather than tooth size assessments which are inherent to conventional techniques. The main contributions providing for holistic and objective morphometric analysis of teeth are the following: (1) interpretation of basic dental morphological features; (2) automated of orientational coordinate system setup based on tooth surface analysis; (3) new tooth morphometric parameters which could not be obtained through application of conventional odontometric techniques; (4) methodological novelty for automated odontomorphometric analysis pipeline. Application of tomographic imaging, which has been used for obtaining 3D models, expands the proposed method potential further through providing detailed and comprehensive reconstructions of teeth. The current study was conducted on unique material from the archaeological site of Sunghir related to the Upper Palaeolithic period. Metric assessments of external and internal morphological layers of teeth were performed in common orientation and sectioning. The proposed technique allowed more profound analysis of Sunghirian teeth which date back to the times of modern human morphology formation.

## 1. Introduction

Studies of dental morphology have a long history as well as are being conducted in different scientific or practical disciplines in our days. Obviously, we should mention dental anthropology as one of the leaders in accumulating detailed knowledge about teeth in various aspects, insofar as such highly mineralized structures as dental and skeletal remains form the basis of study material for physical anthropological research in general [1,2]. Thus, morphological data, including that relating to teeth, would serve for further taxonomical, ethnical, or historical interpretations, which, in their turn, depend to a large extent on study objectives. Of course, in the list of disciplines implementing results of studies of teeth, dentistry is the first among practically oriented ones. However, study approaches and applications of dental morphological data differ in dentistry and physical anthropology. Thus, dentists usually would not focus on detailed studies of morphological varieties to the same extent as expertly trained odontologists. Nevertheless, dentists as well as dental technicians on a daily basis keep an eye on morphology of teeth with regard to their closure and to interaction of opposing dental arches. In addition, such a difference has played a significant part in the process of development of the presented method, which actually has originated from research conducted for studies in dentistry.

In addition, what are the main approaches to dental morphological research? Apparently, visual examination, non-metric assessments, is number one. This is what we start with when opening a classical textbook on dental morphology: description of cusps and grooves separating them or other morphological features, usually classified according to the degree of their expression [3]. In addition, there are reference systems, such as ASUDAS, which allow more organized surfing in the waves of numerous traits. As another example, there is the odontoglyphic method which is based on the studies of groove patterns [4]. Of course, there are other approaches applied to dental research together or separately from visual methods. Thus, a range of studies of teeth (as well as other biological or artificial objects) has been performed through geometric morphometrics, which allows for analyzing shapes. We are not going to expand on other methods as genetic, protein analyses, or radio-carbon dating, however teeth, being material for this type of studies, maintain development of these research directions as well. Nevertheless, special attention within the context of this paper would be paid to measurements of teeth or odontometrics. Most frequently, it has a traditional set of measured distances–mainly two maximal parameters are assessed: mesio-distal and bucco-lingual diameters of tooth crown [5]. Such studies usually require specially trained skills and a manually operated measurer—caliper or odontometer. They are still a conventional part of anthropological research, even though they are more suited for general assessments rather than more or less detailed descriptions of dental morphology. As an example of practical application of odontometry, we can mention measurements conducted on teeth and dental arches as diagnostics in orthodontic treatment planning and post-treatment assessment of the results [6]. Thus, we can see that measurements are widely used for studies in dental research as a method of obtaining objective data for immediate assessments or further calculations. Nevertheless, the principal questions discussed in the current paper are the following: is there is an odontometric technique sufficiently developed to study dental morphology? and, do digital visualization and processing techniques play an essential part in such methods? Without keeping the reader of the article in suspense—the answer is affirmative for both issues; however, it is significantly more important to know why and how, which we are going to clarify in this paper for the method described below—automated digital odontometry (ADO). The importance of the proposed technique is in the introduction of reliable metric approaches in dental research. Thus, a new type of objective data has the potential to reveal unstudied morphological aspects of teeth. This can not only provide for a deeper understanding of dental structure and morphology but has a prospect to be implemented in practical disciplines. It should be emphasized that many diagnostic and technological procedures in dentistry are being conducted in digital format today; moreover, we can expect growth of their shares in dental practice in the future. Thus, numerical data provided by ADO can be more easily integrated in digital techniques dealing with diagnosis, modeling dental crowns, or assessment of treatment results. Forensic dentistry is another discipline which implies use of measurements of teeth and dentures and thus new methods of objective assessments of tooth size, especially those based on analyses of digital 3D reconstructions, can play a substantial role in complicated cases. A whole range of natural sciences, such as anthropology, paleontology, or primatology can get new odontometric data, unprecedented in such features as variety, quantity, density, objectivity, and accuracy. Prerequisites for this are in substantiated interpretation of basic dental morphology and fully automated measurement process, which can create a new vision of dental morphology in taxonomical, evolutionary, and historical aspects. The digital format of measurements provides for a creation of extensive databases of physical parameters of teeth of their parts (enamel thickness, root length, or others), which can be used for research or practice in all of the above-mentioned disciplines.

Imaging techniques give a substantial impetus to development in various disciplines, and this applies both to optical imaging as well as to X-ray based techniques since their invention. Today, these methods have a notable bent towards the digital and developed to a degree of obtaining 3D reconstructions. Of course, odontological studies as well as dental clinical or laboratory technologies have received a significant boost with the introduction of new methodological approaches. This can be compared to some extent with postage service and e-mail; both allow sending texts and documents; however, digital ways effectively cover a major part of correspondence today. Returning to the issue of dental research, in some cases, we can observe reproductions of conventional techniques on digital reconstructions [7]; in others—imaging techniques provide obtaining previously unattainable and detailed data (such as accurate reconstructions of inner dental morphology obtained through micro-CT scanning). The above-mentioned examples are characterized by contribution of digital approaches into morphological studies or technological processes. Thus, the output can be observed in more effective communication between professionals, elimination of several technological stages that are necessary in a traditional workflow with physical objects and materials, or visualization of hidden structures. These pictures of overall development are to a large extent results of advantages introduced by progress in imaging and image processing. However, more interesting results can be expected and achieved when advances in morphological studies and new opportunities provided by imaging and image processing techniques meet each other generating new methodological improvement. In addition, the premise for such collaboration will be described further.

Prior to detailed description of the presented method in further chapters, it is worth mentioning that procedures of imaging, image processing, and 3D reconstruction are initial stages of technological input in dental research. Further improvements are provided by appropriate software based on combination of 2D and 3D image analysis methods with interpretations of basic dental morphology. The current study is not only an introduction of automated digital odontometric technique to a wide audience in the scientific community and researchers. The presented technique has been previously used for measurements conducted separately on external and internal layers of teeth; however, the novelty approach is in a common orientation of enamel and dentine morphological structures which are suggested for enhancing research objectivity. However, this special case is able to describe the potential of automated digital odontometry, which is able to provide methodological support in research, obtaining varieties of diverse odontometric data, which can be applied in odontological studies, practical spheres (modeling teeth) or diagnostics.

## 2. Related Works

### 2.1. Interpretation of Basic Dental Morphology

**Figure 1:**
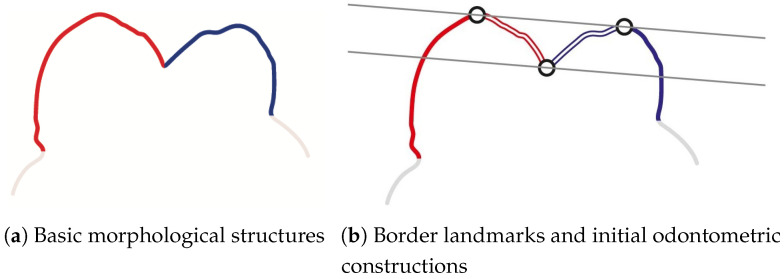
Tooth contour on a transverse section (**a**) with basic morphological structures, border landmarks and initial odontometric constructions (**b**).

Before the development of measurement protocols, it is important to have a corresponding concept of basic dental morphology, as despite variability of teeth, there should be common traits uniting their vast majority. These traits would form the core of the method to provide its usability in a wide range of cases. For instance, teeth have a tendency to change their relief and dimensions as a result of functional wear [8], gradually losing their variety defining features. However, would there be morphological features remaining stable? Thus, the first problem to be resolved is in finding appropriate interpretation of basic dental morphology. An important issue is that human teeth are heterodont, i.e., they differ in morphology, function, and their positions in dental arches. This is a hindering factor for development of a universal approach covering teeth of all groups. Thus, we will focus on posterior teeth that are most numerous in our dentures: premolars and molars; they form variable morphological types within both upper and lower arches, and are very interesting to be studied. Premolars (two on each side of a dental arch) are localized directly beyond canines and usually are composed of two or three cusps. Molars—the most numerous (three on each side of an arch) and the most posterior group of teeth—typically possess from three to five or six cusps. Exceptions are certainly possible, especially in the case of high variability of wisdom teeth. Nevertheless, regardless of morphological details, cusps of teeth can usually be referred either to vestibular (buccal) or to oral (lingual) sides. Thus, as a rule, sectioning of posterior teeth in a transverse (vestibular-oral, or bucco-lingual) direction would give a picture of two elevations—almost an iconic image of tooth contour. Moreover, a lot of studies use exactly the mentioned direction of tooth sections, and numerous morphological interpretations and practical recommendations are presented as such as well. Thus, it should be recognized that the contours discussed are very informative and highly relevant to basic dental morphology (Figure 1a).

In addition, it is evident that the two cusps on these contours can be classified according to their position: vestibular (buccal) and oral (lingual, palatal) either on upper or lower teeth. Actually, difficulties and contradictions start when we give corresponding function to each cusp in this pair (e.g., functional and non-functional cusps, or supporting and guiding cusps; these approaches are commonly encountered in morphological and practical concepts: [9,10,11]. In addition, despite the existence of such concepts, a closer look at their content reveals that the attributed functional difference of cusps is inseparable in fact—each cusp possesses a part of function given to the other cusp, especially when observed with respect to the closure of opposing teeth (and this is exactly what the function implies). Hence, there should be a high probability of morphological support for such close functional linkage observed at different levels of masticatory system organization. In addition, it certainly exists, even on the so familiar tooth contour. However, now it is insufficient to consider only the two cusps—the third morphological structure to be taken into account is the anatomical occlusal surface. It is also very important that these three morphological structures have borders which can be clearly defined: the cusps are separated in the point of maximal deepening, whereas occlusal surface ends at the cusp tips. In addition, these points can be accurately set as far as the contours observed allow construction of only two parallel lines, one of which is tangent to both cusp tips and the other—to the deepest point of occlusal contour (Figure 1b). Thus, this is how the above-mentioned study approaches, which are accepted in dentistry, have provided for setting the basis for the development of measurement techniques presented in the current paper.

### 2.2. Measurement Techniques

Along with different research methods, manually operated measurements are in use today in odontological research, and their results serve for various interpretations; the parameters measured usually remain the same: bucclo-lingual and mesio-distal diameters, which, in their turn, might serve for calculating indices [12,13]. Although there are new, alternative approaches suggested in traditional, typically manually operated measurement techniques as well, e.g., diagonal measurements, cervical measurements, crown heights, etc. [14,15,16].

Application of 3D imaging enhances the potential of odontometrics allowing access to additional measurement data, even in cases of direct application of conventional measurement techniques [17,18]. The choice of imaging method is a factor which can influence the measurements’ results, their accuracy or feasibility of particular parameters measured. Nevertheless reported recommendations for implementing this or that imaging approach can vary from study to study and frequently they are driven by study objectives [19,20,21].

A considerable amount of metric studies in odontology are aimed to assessment enamel thickness as this data are important in biological, palaeontological, and palaeoanthropological research [22,23]. In addition, whereas previously unavoidable physical cross-sectioning of teeth was the preferred approach for this type of studies [24,25], today tomographic imaging allows for preserving the integrity of many unique findings [26,27,28]. Subsequently, the 2D contours obtained through physical or digital sectioning teeth or their 3D reconstructions usually serve for geometric constructions and measurements. However, in some cases, sectioning teeth remains essential for researchers particularly when features of dental tissues have to be studied [29]. It is also noteworthy that studies of two-dimensional contours of teeth have promoted significantly for odontological research in general through providing easier available access to the analysis of the complex shape of teeth.

Orientation is an important issue, especially when it comes to measuring teeth which are complex in shape and in addition possess variable morphology. Manually operated techniques offer detailed instructions for correct positioning of teeth and measurer tips on them; however, it is always a procedure not devoid of subjectivity to an extent. This usually requires several attempts to measuring, re-measuring by other experts, assessments of intra- and inter-observer errors, etc. Digital techniques, in their turn, may contain partially automated orientational stages, such as approximation of plane according to enamel cervical contour [26]. Nevertheless, many of those methods are aimed to perform somewhat ideal orientation, thereby inheriting approaches from physical sectioning, which has serious limitations in the number of successful sectioning attempts or in thickness and number of slices [30]. Beyond that, “3D” orientation of teeth—performed for purposes of setting their cross-sectioning direction [31], there is a necessity to pay attention to proper landmark setting and performing measurement on 2D contours which are actually represented as curved lines. This part can be classified as orientational stage as well—this time on flat surface—“2D” [32].

### 2.3. Imaging Techniques

There are different examples of application of imaging techniques for measuring teeth or dental arches, starting from conventional optical and X-ray flat surface imaging [33,34,35] to more widely used in dental research today 3D reconstructions. 3D imaging for odontological studies includes different approaches as well: from rarely used contact scanning [30] to various types of remote methods. The choice of imaging technique depends to a large extent on the objects studied and reflects study objectives, as most likely, researchers would usually seek higher accuracy and details. Thus, studies of dental casts can be performed by use of photogrammetric equipment [36,37]; various scanners are applied for this type of research [17,38]. However, their lighting parameters usually do not correspond to dental enamel optical features. Thus, they are not intended for scanning teeth unless opaque spray is applied (but it is preferable to avoid contamination either in case of intraoral procedures or for scanning palaeoanthropological findings). As an alternative, there are examples of successful application of intraoral scanners which were initially designed for clinical use in dental practice [39,40,41,42].

In line with optical techniques, imaging teeth has a close link with X-ray based methods, especially with cone beam computed tomography (CBCT) and micro computed tomography (μCT). Despite the fact that CBCT provides less detailed reconstructions of teeth, it remains a widely used imaging method largely due to low dose rate exposure which is important in all medical disciplines. Thus, CBCT is widely in orthodontic research and diagnostics [43,44]. At the same time, this method provides access to inner dental morphology and roots which are concealed under bone tissue [45,46,47]. The increasing reliance on micro-computed tomography, especially in the recent two decades, allows for obtaining accurate and detailed images of outer and inner structures of teeth. These features make μCT applicable in various approaches to studies in palaeoanthropology, palaeontology, biology, or dentistry. This includes such research methods as visual examination [48], geometric morphometric analysis [49,50], measurements [51], or research and education [52]. In general, digital imaging have significantly provided for research and there are numerous cases of their successful application and development of new directions for research [53,54,55,56,57].

## 3. Materials and Methods

The method described in this chapter—automated digital odontometry (ADO)—has previously been applied for research in dentistry, anthropology, and paleontology. Being initially developed only for use with digital reconstructions of teeth, various object types have been involved in studies, including dental casts, separate teeth, skulls, and mandibles, or their fragments, containing teeth (including unerupted). Hence, different imaging techniques have been tested for the method. However, a significant part of recent research applying ADO, including the presented in this paper, has been carried out on palaeoanthropological material, and studies of internal dental morphology (as well as external) become of special interest. These approaches require application of tomographic imaging, providing detailed 3D reconstructions of teeth; certain improvements in measuring technique are also necessary.

### 3.1. Sunghirian Teeth

The current study is performed on palaeoanthropological material which dates back to the Upper Palaeolithic period. Excavations at the archaeological site of Sunghir, situated at the edge of city of Vladimir (Central Russia), started in the late 1950s led by O. Bader [58]. They revealed a settlement, which presumably has been inhabited by hunters as a seasonal camp. Among various findings, there was a tomb which attracted special attention: a paired burial of two laid head-to-head adolescent boys (according to recent genetic analysis—[59]). Numerous artifacts cut of animal bone and mammoth tusks (two long spears, thousands of beads, sculptures, etc.) accompanied discovery of these human remains. Skeletal remains were completely preserved. Regarding odontological research, the denture of elder individual (approximately 11–13 years old; Sunghir 2, or S2) is of special interest not only for its remarkably preserved condition, but for extraordinary morphological features which have been described as archaic [60]; The younger adolescent—S3, of about 9–11 years old, possesses well preserved, though transitional, dentition as well. This state of affairs has already attracted our interest and automated digital odontometry has been implemented for a comparative study of external morphology of Sunghirian teeth and other samples with similar features, though different expression degree up to their complete absence [61]. This and other studies have set directions for further development of automated odontometry, and these methodological improvements have formed the basis for the current research which in fact is an example of simultaneous study of external and internal dental morphology. Thus, the results of measurements conducted on 3D reconstruction of upper left second molar are presented in the following chapters.

### 3.2. Tomographic Scanning

The method of automated digital odontometry has been tested on 3D reconstructions obtained through application of different imaging techniques and in conformity with the studied material. The first attempts to measure dental morphology and occlusion were made after scanning dental casts on the original photogrammetric system [62,63,64]. Recent research has been held on teeth representing palaeoanthropological and palaeontological findings. Thus, in the current study, while choosing the imaging technique, we paid attention to preservation of the unique findings, high accuracy of 3D reconstructions, and access to internal and external dental morphology. We had no serious limitations in terms of X-ray dose exposure for the sample (moreover, genetic and dating studies have been already performed for these findings—[59,65]). Thus, high resolution tomographic scanning would completely correspond to our requirements. In addition, tomography is effective for studies of roots or unerupted teeth (e.g., like wisdom tooth of S2) which can be partially or completely surrounded by bone tissue. However, the studied object is a skull with complete dental aches which had to be preserved in condition of its conservation (removing teeth for more detailed scanning is unacceptable). The appropriate scanner for the object size was Phoenix v|tome|x m (Waygate Technologies (former GE Inspection Technologies), Hurth, Germany) where the skull of S2 individual was centered in order to achieve high accuracy for the posterior teeth as they were of particular interest. Imaging was performed at 275 kV applied to the tube.

### 3.3. Image Processing

After the scanning procedure, images corresponding to each studied tooth were extracted from the entire stack for further processing and obtaining 3D reconstructions, which would allow smooth performance of automated odontometric algorithms. The raw data represented by .BMP format images with 8-bit color depth was processed by Avizo 9.01 software (Thermo Fisher Scientific, Waltham, MA, USA) Segmentation was performed through image thresholding based on difference in absorption levels of such dental and bone tissues as enamel, dentine, cortex, trabecular bone, and the emptiness of pulpal chamber. Subsequently, morphologically irrelevant to dental tissues areas, even though possessing the same grayscale level, were cleaned out; afterwards, 3D reconstruction of teeth was launched. In order to facilitate running of measuring software the reconstructions were processed for surface smoothing, size reduction, and format conversion. Perforations detected on reconstructed surfaces, which could obstruct the measurement process, were “patched” in a manual mode.

**Figure 2:**
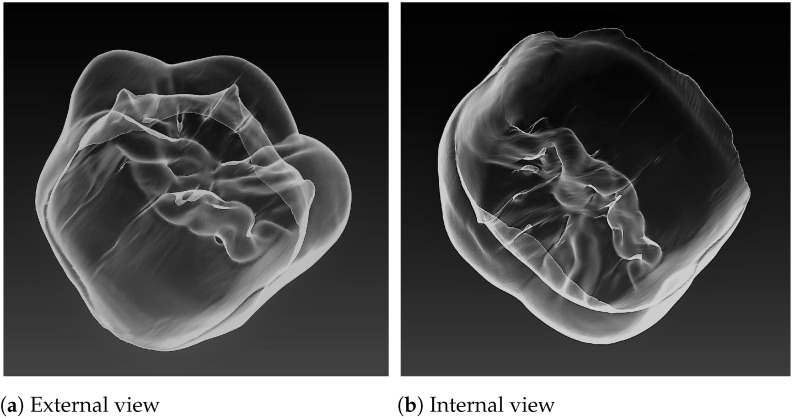
Enamel cap 3D reconstruction in a semi-transparent visualization mode; lower left first molar, S2: external view (**a**) and internal view (**b**).

The combination of such factors as imaging technique and object size allowed for obtaining 3D reconstructions of the Sunghirian teeth (S2) with a voxel size of 43 μm. We were particularly interested in separate models of enamel caps, as they simultaneously depicted on each surface features of external (enamel surface) and congruent to internal (dentine surface) morphology of teeth (Figure 2a,b). Separate studies of internal, composed of dentine, surfaces of teeth are possible as well; however, they have revealed some difficulties with running orientational protocols and surface analysis settings as well as in achieving match of orientations with external (composed of enamel) surface [66]. Thereby, enamel cap reconstruction was used in all stages of measurements in the study, including orientation.

### 3.4. Orientation of Teeth

Orientation is a significant part of the presented method that influences the results obtained through measurements; it is performed automatically in the suggested odontometric technique. The initial part of tooth orientation is based morphologically on the contour of anatomical occlusal surface and serves for vertical axis alignment. This contour actually is the border between centrally located depression on tooth crown and its outer surfaces. It is defined through surface analysis methods and represents a set of points surrounding the occlusal surface.

**Figure 3:**
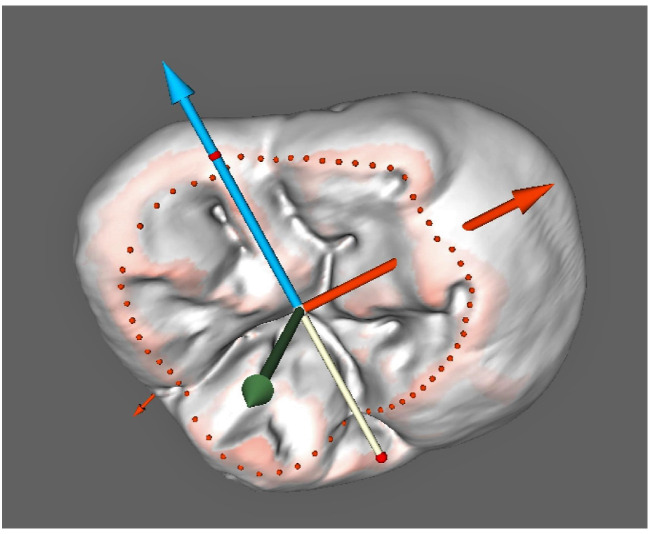
Orientation of axes on S2 upper right unerupted wisdom tooth enamel 3D reconstruction, 30-section mode.

Their coordinates set the position for the center of orientational coordinate system and serve for setting vertical axis inclination, which is marked in green (Figure 3). This process is staged and requires iteration for more accurate settings. We should mention that the use of tomographic imaging allows accurate reconstruction and clear access to morphological structures, which is due to different circumstances can be completely hidden or non-obvious for correct detection. Thus, the edge of cervical enamel has been tested for orientation algorithms in automated digital odontometry in line with occlusal surface contour [67], which we consider a reliable morphological structure as a reference landmark.

The result of the scanning data processing is a 3D model of a tooth T. It is represented in a form of triangulated irregular network (TIN)—a set of coordinates of surface points (nodes) $t_{i},1=1,\cdots N$ and a set of corresponding edges $l_{j},j=1,\cdots L$ that connect the nodes into elementary surface element (facet). To perform correct and interpretable morphometric analysis, it is necessary to define a tooth coordinate system that allows for comparing measuring results for teeth of different shape and dimension. As the occlusal surface seems to be the most relevant reference for teeth morphology comparison, it is taken as a basis for the XY plane of the tooth coordinate system.

The occlusal surface is an area lying inside the occlusal border $O_{b}$ that can be defined as a set of tooth surface points having maximal values of Gaussian curvature $K=\kappa_{1}\times \kappa_{2}$. Here, $\kappa_{1}$ and $\kappa_{2}$ are principal curvatures of a surface. The XY plane is defined by its normal $n_{T}$ that is calculated as an average value of the normals at points of the occlusal border $O_{b}$.

The procedure of the tooth system of coordinates determination is presented as Algorithm 1.
**Algorithm 1:**Tooth system of coordinates determination.
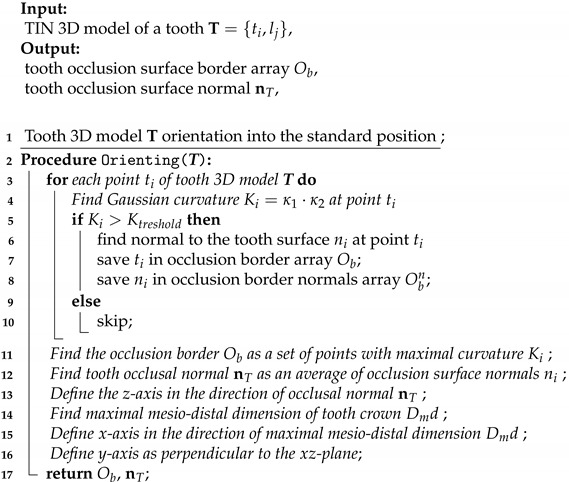


The next stage for orientation is the mesio-distal, or anteroposterior, axis orientation (this axis is marked in two colors: yellow and blue, in order to distinguish its direction easier). It is set perpendicular to the vertical axis and according to the shape of occlusal surface contour.

Additional calibration of mesio-distal axis is performed according to maximal dimensions of tooth crown. The third—vestibulo-oral (bucco-lingual, transverse) axis direction—is set as perpendicular to both mentioned above axes; it is marked in red in Figure 3.

Orientation was performed in the current study for the enamel cap reconstruction, which was studied both in terms of its outer and inner morphological features. Algorithms were set to orientate the system of coordinates according to enamel occlusal surface contour. The same orientation was used for measurements on dentin.

### 3.5. Cross-Sectioning

**Figure 4:**
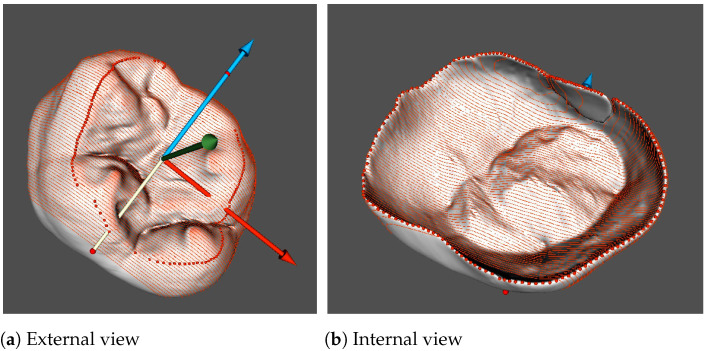
Odontotomy: 80-section mode; upper right second molar, S2; external view (**a**) and internal view (**b**).

Cross-sectioning of teeth, or odontotomy, is performed for obtaining contours, which, in their turn, serve for measurements. In order to obtain the required alignment of transverse contours, the sectioning planes are set perpendicular to the mesio-distal axis (Figure 4). Hence, such contours depict essential features of teeth and allow for carrying out measurements according to morphological interpretation described above.

Sectioning planes are parallel to each other and have equal intervals in-between. The number of sections is a matter of researchers’ choice; however, dense sectioning obviously provides for an increase of measurement accuracy and study objectivity. The sectioning procedure is completely automated.

For tooth sectioning, the occlusal border $O_{b}$ is approximated by an ellipse ϵ, and the sections by $N_{s}$ planes perpendicular to the major axis are generated. In each section, tooth odontometric parameters $\left\{p_{o}^{m}\right\}$ are calculated.

The procedure of the tooth sections generation is presented as Algorithm 2.

The sectioning stage was the same for measuring outer and inner morphology; however, it was performed twice for the 3D reconstruction in the current study: one for the outer, enamel, contour measurements, the other—for inner or dentine. In addition, the 80-section mode was applied for both cases.
**Algorithm 2:** Tooth sections generation
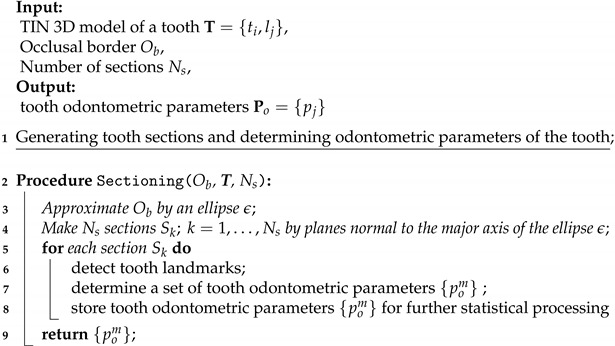


### 3.6. Measurements and Data Collection

The stage of measurements per se is performed on contours obtained through sectioning 3D models of teeth, or like it is presented in the current paper—enamel cap reconstructions. Orientation algorithms are set to display the sections in the frontal view for the researcher, which is achieved by marking the buccal surface of the studied tooth and choosing the tooth number.

**Figure 5:**
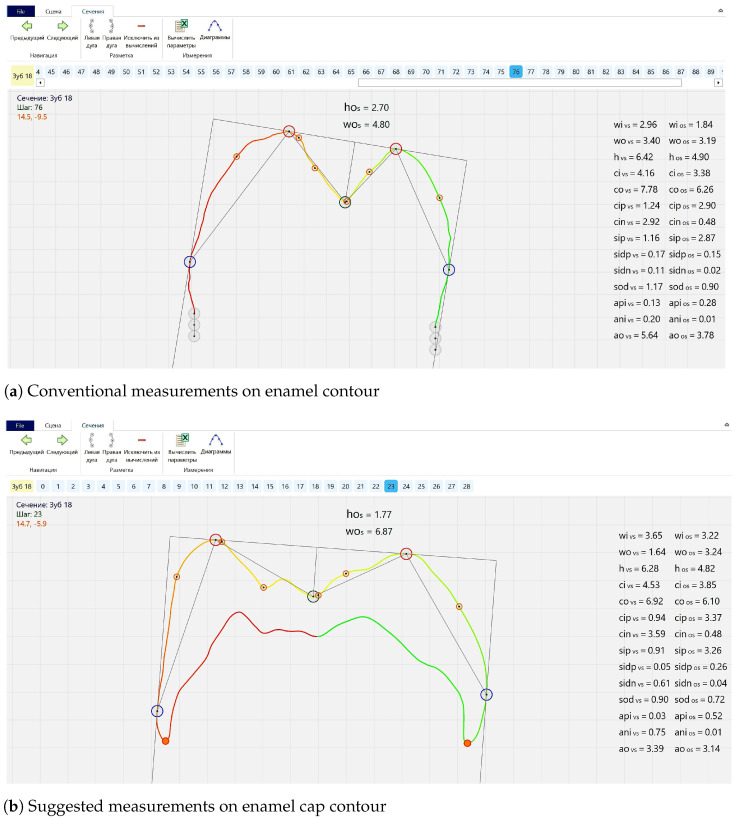
Constructions and landmarks on contours; upper right second molar, S2: conventional measurements on enamel contour (**a**); suggested measurements on enamel cap contour (**b**).

**Figure 6:**
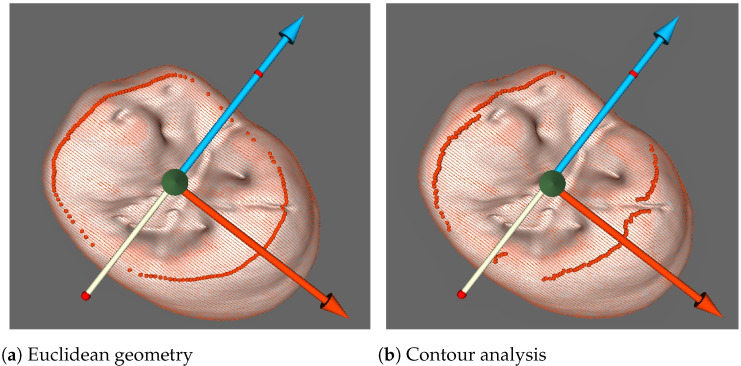
3D view of occlusal surface border landmarks (red dots) set according to Euclidean geometry rules (**a**) and surface contour analysis (**b**); upper right first premolar, S2; 100 section mode.

Conventional measurements, usually using five main landmarks, are based on the described above morphological interpretation. They allow for obtaining odontometric parameters through direct measurements and calculations. These parameters can be classified as linear (vertical and horizontal), angular, contour length and area of surface; coefficients and ratios are calculated as well (Figure 5a). Regarding landmark setting, there are two possible modes to choose: the first completely corresponds to Euclidean geometry rules (Figure 1b); however, the second is based on contour curvature analysis (Figure 6a,b).

As an extension of this technique, we suggest to perform odontometric study simultaneously on contours depicting external (enamel) and internal (dentinal) morphological layers of teeth. In this case, the number of landmarks increases up to twelve (depending on a contour type), including two landmarks mutually shared by both external and internal contours; these landmarks correspond to enamel edges (orange dots in the lower portions of contour on Figure 5b). As a result of such changes, measured parameters can be revised as some of them (e.g., contour lengths or linear vertical parameters) can be set exactly in accordance with cervical margin edges. Moreover, new parameters can be added in the measured range. For instance, shortest distances referring to cervical areas (orange line segment between blue points of maximal external contour convexity and orange points of cervical margin edge—Figure 5b) expand linear and angular parameter list. However, the measurement procedure, in order to facilitate the algorithm performance, was conducted in two stages after separating enamel cap contour in two parts. The separation points are enamel edges on vestibular and oral parts of contours; they divide the enamel cap contour into upper and lower sectors which correspond to enamel and dentine. Curvature analysis mode was used for landmark setting in the current study.

The set of parameters obtained on a single section describes local morphology; for assessment of the overall picture for the tooth, data are collected from the entire set of contours and then average parameters are calculated. The most mesially and distally located marginal sections are usually excluded from data analysis as they can represent distorted morphology or influence average parameters significantly. At the end of the process, all parameters are saved for further analysis.

Thus, 76 enamel and 65 dentin contours out of 80 sections conducted served for measurements. The selection criterions were complete depiction of dental morphology and the absence of imperfections on contours (such as downed lines). Thus, marginal sections were excluded, and dentine, being more compact in volume than enamel, could be visualized on a lesser number of sections. All mentioned contours were analyzed; however, only several linear parameters are presented in the article, without expanding on complete morphological features which are provided by the method applied.

### 3.7. Automation

The automation degree can be estimated as high in the suggested technique; actually, it is complete, however pauses are included for assessment of proper algorithm execution. Thus, at the end of each stage: orientational, sectioning, and measuring, operator can re-adjust settings or correct intermediate results manually if necessary. However, there is an opportunity to choose settings prior to measurements or re-measurements in the case of inadequate algorithm performance. For instance, curvature radius for surface analysis or number of sections can be set in advance. The measurement software suggests tools for correction of orientational axes inclination as well as for occlusal surface contour outlines; landmarks’ positions can be changed on flat contours as well.

This study was conducted in fully automated modes partly because of necessity of reloading the 3D reconstruction for measuring dentine. Dentine measurement mode was chosen as the second round of orientation. No adjustments were required for orientation in 3D mode; only landmark position was corrected on one of 2D dentine contours.

## 4. Results

The current study is an example of metric assessment of dental morphology performed by application of an automated digital odontometric method. The measurements were conducted on 3D models of teeth, to be exact—their enamel caps, representing external and internal morphology of coronal parts. Teeth reconstructions, which refer to Upper Palaeolithic findings of Sunghir, were obtained through micro tomographic imaging. Measuring procedure was performed on 2D contours of enamel and dentine after similar for both orientating and sectioning stages.

Feasible 3D data processing software, such as 3D Slicer or ITK Snap, can be used to perform procedures needed, but mostly in manual mode, thus requiring a vast amount of time for extracting needed morphological features. Our original software performs all key procedures (such as occlusal surface detection, tooth orientation, tooth sectioning, morphological parameters extraction) in automated mode, thus giving more time for an expert for extracted features analysis.

Available publications [68,69,70] on comparative accuracy of CBCT, μCT and other 3D scanning techniques reported that μCT is the preferred approach in odontological research; CBCT reconstructions are inconsistent in areas that are of importance in the proposed measurement technique, especially when larger objects are scanned. Our previous research [42] and these publications allow us to choose μCT as the most accurate and the most adequate mean for our study.

The linear parameters presented in Table 1 refer to tooth coronal part and its occlusal surface relief. They characterize dimensions of tooth and its anatomical occlusal surface in vertical and vestibular-oral (bucco-lingual) directions. Results are presented in a similar way to a traditional manual measurement technique—as maximal values (their positions in the studied sequence of slices is a matter of interest as well). In addition, average values which were calculated as arithmetic mean of parameters, which were obtained on the whole set of contours, are presented in the same table.

Maximal vestibular-oral dimensions of teeth show a high degree of consistency when measured on enamel and dentine not only in terms of their absolute values (which is, to a large extent, due to cervical—for dentine and very close to cervical—for enamel positions of measuring points), but in terms of localisation as well. Thus, sections 43 for enamel and 40 for dentine show maximal parameters; both correspond to the median portion of tooth crown in case of 80-section odontotomy. The same parameters measured for occlusal surface show a lower degree of similarity in terms of absolute values (10.07 mm for enamel and 7.76 mm for dentine), which is due to thicker enamel on an occlusive area than at the cervix. Nevertheless, sections with maximal vestibular-oral dimensions of occlusal surface are localized close to each other on enamel and dentine (sections number 33 and 28 respectively) and have distal displacement. This is partly explained by algorithm settings which are influenced by pronounced deep and distally localized palatal groove on this tooth. It is remarkable that maximal and average measurements in vestibular-oral direction on this tooth show consistency which can not be observed on vertical parameters. Occlusal surface is significantly deeper on enamel than on dentine which can be observed on maximal and average parameters. Moreover, sections with maximal vertical values are localized differently: in the mesial third on dentine (section number 50) and distally on enamel (section number 17).

Parameters measured and calculated allow analysis of morphological features of tooth. Thus, ratios of vestibular-oral dimensions of tooth crown and its occlusal surface as well as ratios of vertical dimensions of occlusal surface are presented. An important feature of the current study is that the results refer to enamel and dentine surfaces, and the parameters obtained serve for comparisons of these two morphological layers within a tooth. Thus, we suggest here, as an example of possible comparative analysis, calculation of percentage of dentine parameters with respect to enamel parameters, which are presented in Table 2.

Similarly to absolute values, ratios calculated for the parameters, which were measured in vestibular-oral direction, possess a higher degree of consistency if compared with vertical parameters. It is interesting though that ratios of average parameters on enamel and dentin have a tendency to exceed slightly those for maximal values in case of vestibular-oral measurements (96.78% and 77.06%—maximal vs. 97.82% and 80.76%—average). The opposing is observed on vertical maximal and average percentage values (64.24% vs. 59.27%). As another approach to data analysis we can suggest ratios of vertical and horizontal (vestibular-oral) parameters of occlusal surface. They are calculated for average parameters as vertical and horizontal maximums are registered on different sections, hence comparisons are not correct. In addition, according to the values obtained, we can conclude that occlusal surface is shallower on dentine (0.19) than on enamel (0.27) on the studied tooth.

We will not expand further on odontometric data in this article. However, it is necessary to mention that the linear measurements presented are only a small portion of the parameter variety, which becomes accessible through application of automated digital odontometry. In addition, for the tooth mentioned above and occlusal surface assessments, more morphological structures can be measured, such as cusps or cusp slopes.

## 5. Discussion

Automated digital odontometry (ADO) was applied in its latest version for measurements in the current study. In addition, this method, due to features which will be discussed below, has a potential to improve odontological research in different directions. Thus, if viewed in terms of odontometric studies, the suggested approach expands their potential from generalized assessment of tooth size to capability of detailed morphological studies—whereas, from positions of morphological research, ADO can complement non-metric methods through providing accurate, obtained through measurements and thereby objective data (it can be used in geometric morphometrics as well).

In comparison with conventional manual measurements, application of automated digital odontometry provides a significantly larger amount and a variety of dense and evenly distributed across the studied tooth data. In addition, the method performs rapidly and does not require operators’ interference (nevertheless, it allows adjustment of settings and control over the process). However, the key role in the method usability should be given to combination of two components: interpretation of basic dental morphology and application of imaging and image analysis techniques. The first sets rules for development of measurement protocols, which possess potential to cover a significant part of variety of teeth and to identify morphological structures that would support orientation and measurements. At the same time, these rules should correspond to geometric principles in order to provide uniformity of measurements and correctness of dental morphology assessments on separately studied teeth or in comparative studies—within a group of samples. In this connection, it should be noted that application of the approaches described above to physical objects—teeth—would be difficult. At least such a technique, possessing similar features as ADO, would be very laborious, time-consuming, and definitely completely destructive for studied objects—whereas employing digital techniques and methods of surface analysis allows implementation of the suggested morphological interpretations and protocols developed on their bases on 3D reconstructions of teeth. Hence, application of digital imaging and image analysis techniques are the obligatory requirements for the proposed approach to odontological research and for providing corresponding outcomes.

The importance and use of imaging techniques in anthropological, palaeontological, and odontological research, or in dental technology, become an increasing trend, or, as for the discussed study method in particular—an essential, indispensable integral part. Micro computed tomography (μCT) has proved to be an appropriate imaging technique for the current study. In terms of accuracy, it can compete with various scanners applied for odontological research or involved in dental technological workflow, at the same being more advanced than cone beam computed tomography (CBCT) in the details of obtained reconstructions. In addition, it is worth mentioning that these details are the studied matters in dental morphological research in general and in the presented technique in particular. Nevertheless, reading through reviews, differently interpreting CBCT usability for metric studies, this technique has to be taken as effectively safe for in vivo imaging purposes. However, an imaginary technique that would combine features of CBCT low dose rate exposure with μCT accuracy level could be effective and informative for dental research in the future, e.g., for tracking functional morphological changes or in diagnostics referred to conditions caused by closure of opposing teeth. In addition, a far greater number of samples could be studied, thereby improving the level of overall understanding of variable dental morphology. Discussing X-ray based techniques, it is necessary to mention their important feature—the ability of imaging internal, as well as external, morphological layers of teeth, providing essential data for supporting modern methods of research. However, the choice of an appropriate imaging technique requires consideration of physical features of studied objects, which means the application of different methods, such as optical scanning for dental casts.

3D reconstructions obtained through micro computed tomography provide accurate data referring to morphology and shape of studied teeth; however, the measurement stage is performed on two-dimensional contours. As a matter of fact, such contours have promoted significantly, for odontological research in general, through facilitating analysis of complexities of dental morphology and making them more accessible. In addition, when it comes to sectioning teeth or their 3D reconstructions for obtaining contours, orientation becomes an important issue. For instance, pilot studies have showed that even 0.5 mm displacements of one end of mesio-distal orientational axis has a marked impact on measured parameters; two or three sequential displacements change them significantly.

The application of 3D reconstructions allows for performing orientation objectively—according to algorithms based on morphological features of teeth which can be clearly detected through surface analysis techniques. We suggest anatomical occlusal surface as a reliable morphological structure and a reference landmark for orientational purposes. Even though its contour outlines and relief are subjected to changes throughout our lives due to masticatory function, occlusal surface still preserves its presence and, in addition, it reflects the mentioned changes. Thus, being a structure possessing morphological and functional importance, occlusal surface plays a significant part in methodological substantiation and in the measurement technique per se, in particular—in orientation.

Subsequent to the stage of orientation comes sectioning, or odontotomy. This study approach is widely practiced in odontology; as a rule, one or two sections are obtained for measurements on depicted contours. However, in the method proposed, we suggest implementation of multi-sectional odontotomy which contributes to study objectivity, as evenly distributed data improve the potential of metric assessments. For instance, maximal parameters, measured in ADO studies with respect to traditional odontometric approaches, are picked from a range of properly orientated sections, hence avoiding measurer position adjustment at this stage. In addition, average parameters are calculated; they refer to the whole tooth or to its parts (cusps, cusp slopes, anatomical occlusal surface), which gives a truer picture than maximal values. The mentioned averages are arithmetic mean values and can be calculated for all parameters measured on a set of sections. Measuring software provides choice in number of sections obtained, which is limited to one hundred units now. Usually, we prefer to use maximal sectioning for our studies; however, there are plans of setting a recommended number of slices. Trials of their reduction have revealed that there are limits impacting the obtained data, including maximal and average parameters.

An important feature of tomographic imaging is in lifting up the lid covering internal dental structures, which can now be studied and compared to external morphology. Our previous studies include measurements conducted separately on enamel and dentine reconstructions; however, comparisons are a subject of interest, especially taking into consideration differences in changes that occur on external and internal morphological layers of teeth. For instance, dentine has higher chances to preserve intact morphology, even when enamel shows signs of mild wear. Nevertheless, it should be noted that there are difficulties with running orientational protocols with the same settings on enamel and dentine due to differences in their reliefs. In addition, there are technical hitches with combining two different orientations in order to achieve correct conditions for comparisons (when two different models of enamel and dentin of one tooth are studied). Thus, currently conducted automated digital odontometry is an example of simultaneous study of both external and internal layers—after one and common for both orientation and sectioning. Such methodological improvement allows for expediting the measurement process, however, which can be assumed more important, provides for measurement objectivity. Digital techniques provide freedom and flexibility in the process of research which are unattainable on physical objects, e.g., re-orientation and new slicing are possible even after obtaining measurement results, whereas tomographic imaging expands opportunities of orientational algorithms providing reconstruction of more comprehensive morphological details, at the same time providing access to intact morphology of unerupted teeth.

Measurements, conducted on combined contours of enamel and dentine, require setting landmarks determining geometric constructions and defining measured distances. It is analogous to orientation, but this time conducted on 2D tooth contours. The landmark setting, which starts from localizing three principal points according to the proposed morphological interpretation, has two possible modes of setting the landmark position. The first completely corresponds to Euclidean geometry rules (Figure 1b); however, the second is based on contour curvature analysis. It is interesting to observe the difference of performing these two approaches visualized in 3D in order to recognize that dental morphology is not exactly Euclidean; however, it is possible to follow strict rules and protocols as well (Figure 6a,b). In both cases of landmark setting, when tomographic imaging is used, 2D contours show cervical edges of enamel. This means additional landmarks and expanding the number of parameters which can be potentially measured and analyzed for more profound analysis of dental morphology. This thereby sets the direction for the near future improvements of automated digital odontometry as a method of dental morphological research. In addition, the mentioned cervically located landmarks helped us to set contour analysis algorithms which we used in the current software version. They served as points for external and internal contour separation which was used for avoiding measurements on contours which are overloaded by an excess of geometric constructions.

The measured parameters, even though related to a very limited part of potentially available range, represent maximal values. They provide an opportunity of odontometric assessments which have a lot in common with conventional techniques. In addition, and in their turn, average values obtained from dense and evenly distributed across the whole tooth measurements, are appropriate for more comprehensive morphometric studies, detailed analysis, and comparisons. We would not put here a detailed discussion of odontometric aspects related to the parameters measured; however, there is a point that is important to highlight. It is the observed higher degree of consistency in parameters obtained in the vestibular-oral direction if compared to vertical measurements. This can be additional evidence of correctness of morphological interpretations forming the basis of the suggested technique, which we are planning to improve using appropriate imaging techniques and image processing for further applications in dental research.

## 6. Conclusions

The proposed technique has been developed for morphometric analysis of teeth. It is based on the presented interpretation of dental morphology, which forms the direction for fully automated pipeline including stages of orientation of coordinate system for the teeth studied, subsequent sectioning according to the obtained orientation and further extraction of morphometric parameters. The method provides for ensuring measurement objectivity and integrity of comparisons of morphological features of enamel and dentine, which were studied in the article presented.

Setting of the tooth coordinates system in the suggested technique is performed through surface analysis using the morphological structure of anatomical occlusal surface as reference; this provides a common approach even in a wide morphological variety of teeth. The measurements are conducted on a set of contours, which are obtained after sectioning teeth, through automated detection of landmarks; some of the parameters are calculated.

The proposed method of automated digital odontometry allows for extracting new parameters for comprehensive studies of dental morphology. Thus, the technique has been applied for morphometric study of tomographically obtained reconstructions of teeth from the archaeological site of Sunghir related to the Upper Palaeolithic period. This allows for studying new features related to periods of formation of modern human morphology.

## Figures and Tables

**Table 1:** Odontometric parameters for enamel and dentine, upper left second molar, S2.

	2.7 Enamel, S2	2.7 Dentine, S2
	vestibular-oral maximal (section number), mm	
tooth	13.98 (43)	13.53 (40)
occlusal surface	10.07 (33)	7.76 (28)
	vestibular-oral average, mm	
tooth	12.26	12.01
occlusal surface	7.13	5.75
	vertical maximal (section number), mm	
occlusal surface	3.02 (17)	1.94 (50)
	vertical average, mm	
occlusal surface	1.93	1.14

**Table 2:** Proportions of dentine and enamel parameters, upper left second molar, S2.

2.7, S2	
vestibular-oral maximal	
tooth; dentine, %	96.78
occlusal surface, dentine, %	77.06
vestibular-oral average	
tooth; dentine, %	97.82
occlusal surface, dentine, %	80.76
vertical maximal	
occlusal surface, dentine, %	64.24
vertical average	
occlusal surface, dentine, %	59.27
vertical/horizontal (vestibular-oral), average	
occlusal surface, enamel	0.27
occlusal surface, dentine	0.19

## Data Availability

Data available on request due to restrictions. The data presented in this study are available on request from the corresponding author. The data are not publicly available due to the policy of the owner.

## Abbreviations

The following abbreviations are used in this manuscript:

ADO	Automated Digital Odometry
CBCT	Cone Beam Computed Tomography
CT	Computed Tomography
μCT	Micro-Computed Tomography
TIN	Triangulated Irregular Network

## References

[1] Rathmann H., Reyes-Centeno H., Ghirotto S., Creanza N., Hanihara T., Harvati K. (2017). Reconstructing human population history from dental phenotypes. Sci. Rep..

[2] de Castro J.M.B., Martinón-Torres M., de Pinillos M.M., Campos C.G., Modesto-Mata M., Martín-Francés L., Arsuaga J.L. (2018). Metric and morphological comparison between the Arago (France) and Atapuerca-Sima de los Huesos (Spain) dental samples, and the origin of Neanderthals. Quat. Sci. Rev..

[3] Hillson S. (1996). Dental Anthropology.

[4] Zoubov A.A., Albert A.D., Thomas M.G. (2011). Odontoglyphics: The Laws of Variation of the Human Molar Crown Microrelief. Orofacial Growth and Development.

[5] Berger L.R., Hawks J., Ruiter D.J., Churchill S.E., Schmid P., Williams S., DeSilva J., Kivell T.L., Skinner M., Musiba C. (2015). *Homo naledi*, a new species of the genus Homo from the Dinaledi Chamber, South Africa. eLife.

[6] Machado V., Botelho J., Mascarenhas P., Mendes J.J.B., Delgado A. (2019). A systematic review and meta-analysis on Bolton’s ratios: Normal occlusion and malocclusion. J. Orthod..

[7] Camardella L.T., Breuning H., Vilella O.D.V. (2017). Are there differences between comparison methods used to evaluate the accuracy and reliability of digital models?. Dent. Press J. Orthod..

[8] Farzin M., Giti R., Heidari E. (2020). Age-related changes in tooth dimensions in adults in Shiraz, Iran. J. Int. Oral Health.

[9] Shillingburg H.T. (1976). Conservative preparations for cast restorations. Dent. Clin. N. Am..

[10] Rosentiel S., Land M., Fujimoto J. (2006). Contemporary Fixed Prosthodontics.

[11] Kraus B.S., Jordan R.E., Abrams L. (1992). Dental Anatomy and Occlusion.

[12] Nakayama M., Kondo O., Pesonen P., Alvesalo L., Lähdesmäki R. (2018). Influence of long and short arms of X chromosome on maxillary molar crown morphology. PLoS ONE.

[13] Kaifu Y., Kono R., Sutikna T., Saptomo W.E., Due Awe R. (2015). Unique Dental Morphology of Homo floresiensis and Its Evolutionary Implications. PLoS ONE.

[14] Hillson S., FitzGerald C., Flinn H. (2005). Alternative Dental Measurements: Proposals and Relationships With Other Measurements. Am. J. Phys. Anthropol..

[15] Pilloud M., Hillson S. (2012). Brief communication: The use of alternative dental measurements on deciduous teeth. Am. J. Phys. Anthropol..

[16] Peckmann T., Meek S., Dilkie N., Mussett M. (2015). Sex Estimation Using Diagonal Diameter Measurements of Molar Teeth in African American populations. J. Forensic Leg. Med..

[17] Smith R., Zaitoun H., Coxon T., Karmo M., Kaur G., Townsend G., Harris E., Brook A. (2009). Defining new dental phenotypes using 3Dimage analysis to enhance discrimination and insights into biological processes. Arch. Oral Biol..

[18] Sassani S., Patel D., Farella M., Henneberg M., Ranjitkar S., Yong R., Swindells S., Brook A.H. (2018). Variation in tooth crown size and shape are outcomes of the complex adaptive system associated with the tooth number variation of hypodontia. Int. J. Des. Nat. Ecodynamics.

[19] Naidu D., Freer T. (2013). The evidence supporting methods of tooth width measurement: Part II. Digital models and intra-oral scanners. Aust. Orthod. J..

[20] Paredes V., Tarazona B., Zamora N., Hern Y., San J.V. (2020). A comparative study of reproducibility, reliability and accuracy in measuring mesiodistal tooth sizes using four different methods—2D digital, 3D CBCT, and 3D CBCT segmented and 3D intraoral scanner. Issues of Contemprorary Orthodontics.

[21] Correia G.D.C., Habib F.A.L., Vogel C.J. (2014). Tooth-size discrepancy: A comparison between manual and digital methods. Dent. Press J. Orthod..

[22] Martin L.B. (1983). The Relationships of the Later Miocene Hominoidea. Ph.D. Thesis.

[23] Zanolli C., Bayle P., Bondioli L., Dean M.C., Le Luyer M., Mazurier A., Morita W., Macchiarelli R. (2017). Is the deciduous/permanent molar enamel thickness ratio a taxon-specific indicator in extant and extinct hominids?. Comptes Rendus Palevol.

[24] Grine F. (2004). Enamel thickness of deciduous and permanent molars in modernHomo sapiens. Am. J. Phys. Anthr..

[25] Olejniczak A.J., Gilbert C.C., Martin L.B., Smith T.M., Ulhaas L., Grine F.E. (2007). Morphology of the enamel-dentine junction in sections of anthropoid primate maxillary molars. J. Hum. Evol..

[26] Benazzi S., Viola B., Kullmer O., Fiorenza L., Harvati K., Paul T., Gruppioni G., Weber G.W., Mallegni F. (2011). A reassessment of the Neanderthal teeth from Taddeo cave (southern Italy). J. Hum. Evol..

[27] Smith T.M., Houssaye A., Kullmer O., Le Cabec A., Olejniczak A.J., Schrenk F., De Vos J., Tafforeau P. (2018). Disentangling isolated dental remains of Asian Pleistocene hominins and pongines. PLoS ONE.

[28] Jones M.E.H., Lucas P.W., Tucker A.S., Watson A.P., Sertich J.J.W., Foster J.R., Williams R., Garbe U., Bevitt J., Salvemini F. (2018). Neutron scanning reveals unexpected complexity in the enamel thickness of an herbivorous Jurassic reptile. J. R. Soc. Interface.

[29] Hu R., Zhao L. (2020). Molar crown formation times of fossil orangutan molars from Guangxi, China. Am. J. Phys. Anthr..

[30] Benazzi S., Fantini M., De Crescenzio F., Persiani F., Gruppioni G. (2009). Improving the spatial orientation of human teeth using a virtual 3D approach. J. Hum. Evol..

[31] Benazzi S., Panetta D., Fornai C., Toussaint M., Gruppioni G., Hublin J.-J. (2013). Technical Note: Guidelines for the digital computation of 2D and 3D enamel thickness in hominoid teeth. Am. J. Phys. Anthr..

[32] Suwa G., Kono R.T. (2005). A Micro-CT Based Study of Linear Enamel Thickness in the Mesial Cusp Section of Human Molars: Reevaluation of Methodology and Assessment of Within-Tooth, Serial, and Individual Variation. Anthropol. Sci..

[33] Kazmi S., Anderson P., Liversidge H. (2017). A Radiographic Study of Pulp Crown Dimensions of the Mandibular Deciduous Second Molar. Int. J. Dent. Oral Health.

[34] Devulapalli R.V., Nadendla L.K., Paramkusam G., Pokala A. (2016). Identification of gender using radiomorphometric measurements of canine by discriminant function analysis. Indian J. Dent. Res..

[35] Tarazona B., Llamas J., Cibrian R., Franco J., Paredes V. (2011). A comparison between dental measurements taken from CBCT models and those taken from a Digital Method. Eur. J. Orthod..

[36] Knyaz V., Zheltov S., Gabuchyan A., Bolshakov G. (2007). Photogrammetric system for automated teeth arches 3D models generation and teeth occlusion analysis. Optical 3D Measurement Techniques VIII.

[37] Arapovic-Savic M., Savic M., Umicevic-Davidovic M., Arbutina A., Nedeljkovic N., Glisic B. (2019). A novel method of photogrammetry measurements of study models in orthodontics. Srp. Arh. Za Celok. Lek..

[38] Jacquet W., Nyssen E., Ibel G.V., Vannet B. (2013). On the augmented reproducibility in measurements on 3D orthodontic digital dental models and the definition of feature points. Aust. Orthod. J..

[39] Jacob H.B., Wyatt G.D., Buschang P.H. (2015). Reliability and validity of intraoral and extraoral scanners. Prog. Orthod..

[40] Abduo J., Elseyoufi M. (2018). Accuracy of Intraoral Scanners: A Systematic Review of Influencing Factors. Eur. J. Prosthodont. Restor. Dent..

[41] Logozzo S., Zanetti E., Franceschini G., Kilpelä A., Mäkynen A. (2014). Recent advances in dental optics—Part I: 3D intraoral scanners for restorative dentistry. Opt. Lasers Eng..

[42] Gaboutchian A.V., Knyaz V.A. (2019). 3D images for automated digital odontometry. Int. Arch. Photogramm. Remote Sens. Spatial Inf. Sci..

[43] Hernandez Y., Tarazona B., Zamora N., Cibrián R., Gandia J.L., Paredes V. (2015). Comparative study of reproducibility and accuracy in measuring mesiodistal tooth sizes using three different methods: 2D digital, 3D CBCT, and 3D CBCT segmented. Oral Radiol..

[44] Ferreira J.B., O Christovam I., Alencar D.S., Da Motta A.F.J., Mattos C.T., Saramago A. (2017). Accuracy and reproducibility of dental measurements on tomographic digital models: A systematic review and meta-analysis. Dentomaxillofacial Radiol..

[45] Afify M., Salem W., Mahmoud N. (2019). Age Estimation from Pulp/Tooth Area Ratio of Canines using Cone-Beam Computed Tomography Image Analysis: Study of an Egyptian Sample. J. Forensic. Res..

[46] Shinde G.R., Mhaisekar R.D., Chaube S.H., Barad A.N., Bhadange S., Patel H.J. (2019). Assessment of Correlation of Growth Hormone Receptor Gene with Tooth Dimensions: A CBCT and Genotyping Study. J. Pharm. Bioallied Sci..

[47] Hikita Y., Yamaguchi T., Tomita D., Adel M., Nakawaki T., Katayama K., Maki K., Kimura R. (2018). Growth hormone receptor gene is related to root length and tooth length in human teeth. Angle Orthod..

[48] Skinner M.M., Evans A., Smith T.M., Jernvall J., Tafforeau P., Kupczik K., Olejniczak A., Rosas A., Radovcić J., Thackeray F. (2010). Brief communication: Contributions of enamel-dentine junction shape and enamel deposition to primate molar crown complexity. Am. J. Phys. Anthr..

[49] Skinner M.M., Gunz P., Wood B.A., Hublin J.-J. (2009). How Many Landmarks? Assessing the Classification Accuracy of Pan Lower Molars Using a Geometric Morphometric Analysis of the Occlusal Basin as Seen at the Enamel-Dentine Junction. Comp. Dent. Morphol..

[50] Krenn V.A., Fornai C., Wurm L., Bookstein F.L., Haeusler M., Weber G.W. (2019). Variation of 3D outer and inner crown morphology in modern human mandibular premolars. Am. J. Phys. Anthr..

[51] Smith T.M., Olejniczak A., Zermeno J.P., Tafforeau P., Skinner M., Hoffmann A., Radovčić J., Toussaint M., Kruszynski R., Menter C. (2012). Variation in enamel thickness within the genus Homo. J. Hum. Evol..

[52] Kato A., Ziegler A., Utsumi M., Ohno K., Takeichi T. (2016). Three-dimensional imaging of internal tooth structures: Applications in dental education. J. Oral Biosci..

[53] Weber G.W., Bookstei F.L. (2011). Virtual Anthropology—A Guide to a New Interdisciplinary Field.

[54] Uldin T. (2017). Virtual anthropology—A brief review of the literature and history of computed tomography. Forensic Sci. Res..

[55] Chen H., Zhang K., Lyu P., Li H., Zhang L., Wu J., Lee C. (2019). A deep learning approach to automatic teeth detection and numbering based on object detection in dental periapical films. Sci. Rep..

[56] Kniaz V.V., Mizginov V., Papazyan A., Fomin N., Grodzitsky L. (2021). Adversarial Dataset Augmentation Using Reinforcement Learning and 3D Modeling. Studies in Computational Intelligence.

[57] Chen Q., Huang J., Salehi H.S., Zhu H., Lian L., Lai X., Wei K. (2021). Hierarchical CNN-based occlusal surface morphology analysis for classifying posterior tooth type using augmented images from 3D dental surface models. Comput. Methods Programs Biomed..

[58] Bader O.N. (1959). Palaeolithic Settlements of Sunghir on the Klyazma River. Soviet Archaeology.

[59] Sikora M., Seguin-Orl O.A., Sousa V.C., Albrechtsen A., Korneliussen T., Ko A., Rasmussen S., Dupanloup I., Nigst P.R., Bosch M.D. (2017). Ancient genomes show social and reproductive behavior of early Upper Paleolithic foragers. Science.

[60] Zubov A.A. (1984). Morphological Study of Childrens’ Teeth from the Sughirian Burial Sunghir.

[61] Gaboutchian A.V., Knyaz V.A., Leybova N.A., Simonyan H.Y., Novikov M.M., Apresyan S.V., Cherebylo S.A., Petrosyan G.R. (2016). 3D reconstruction and image processing of anthropological archaeological findings. Int. Arch. Photogramm. Remote Sens. Spatial Inf. Sci..

[62] Knyaz V.A. (2012). Image-based 3D reconstruction and analysis for orthodontia. ISPRS Int. Arch. Photogramm. Remote. Sens. Spat. Inf. Sci..

[63] Knyaz V.A. (2015). Scalable photogrammetric motion capture system “mosca”: Development and application. Int. Arch. Photogramm. Remote Sens. Spatial Inf. Sci..

[64] Knyaz V.A., Gaboutchian A.V. (2016). Photogrammetry-based automated measurements for tooth shape and occlusion analysis. Int. Arch. Photogramm. Remote Sens. Spatial Inf. Sci..

[65] Sulerzhitskiy L.D., Petit P., Bader O.N. (2000). Radiouglerodniy vozrast poseleniya i obnaruzhennykh pogrebeniy. Homo Sungerensis, Verkhnepaleoliticheskiy Chelovek: Evolutsionnye i Ekologicheskiye Aspekty Issledovaniya.

[66] Gaboutchian A.V., Knyaz V.A., Novikov M.M., Vazyliev S.V., Korost D.V., Cherebylo S.A., Kudaev A.A. (2021). Comparative morphological analysis of enamel and dentin surfaces’ reconstructions by means of automated digital odontometry. ISPRS Int. Arch. Photogramm. Remote. Sens. Spat. Inf. Sci..

[67] Gaboutchian A.V., Knyaz V.A., Vasilyev S.V., Korost D.V., Kudaev A.A. (2021). Orientation vs. orientation: Image processing for studies of dental morphology. ISPRS Int. Arch. Photogramm. Remote. Sens. Spat. Inf. Sci..

[68] Taylor T.T., Gans S., Jones E.M., Firestone A.R., Johnston W., Kim D.-G. (2013). Comparison of micro-CT and cone beam CT-based assessments for relative difference of grey level distribution in a human mandible. Dentomaxillofacial Radiol..

[69] Maret D., Peters O., Galibourg A., Dumoncel J., Esclassan R., Kahn J.-L., Sixou M., Telmon N. (2014). Comparison of the Accuracy of three-dimensional Cone-beam Computed Tomography and Micro–Computed Tomography Reconstructions by Using Different Voxel Sizes. J. Endod..

[70] Liang X., Zhang Z., Gu J., Wang Z., Vandenberghe B., Jacobs R., Yang J., Ma G., Ling H., Ma X. (2017). Comparison of micro-CT and cone beam CT on the feasibility of assessing trabecular structures in mandibular condyle. Dentomaxillofacial Radiol..

